# Use of the Fitbit to Measure Adherence to a Physical Activity Intervention Among Overweight or Obese, Postmenopausal Women: Self-Monitoring Trajectory During 16 Weeks

**DOI:** 10.2196/mhealth.4229

**Published:** 2015-11-19

**Authors:** Lisa Cadmus-Bertram, Bess H Marcus, Ruth E Patterson, Barbara A Parker, Brittany L Morey

**Affiliations:** ^1^Department of KinesiologyUniversity of Wisconsin - MadisonMadison, WIUnited States; ^2^Department of Family Medicine and Public HealthUniversity of California, San DiegoLa Jolla, CAUnited States; ^3^Department of MedicineUniversity of California, San DiegoLa Jolla, CAUnited States

**Keywords:** exercise, health behavior, health promotion, Internet, mHealth, motor activity, physical activity, technology, women

## Abstract

**Background:**

Direct-to-consumer trackers and devices have potential to enhance theory-based physical activity interventions by offering a simple and pleasant way to help participants self-monitor their behavior. A secondary benefit of these devices is the opportunity for investigators to objectively track adherence to physical activity goals across weeks or even months, rather than relying on self-report or a small number of accelerometry wear periods. The use of consumer trackers for continuous monitoring of adherence has considerable potential to enhance physical activity research, but few studies have been published in this rapidly developing area.

**Objective:**

The objective of the study was to assess the trajectory of physical activity adherence across a 16-week self-monitoring intervention, as measured by the Fitbit tracker.

**Methods:**

Participants were 25 overweight or obese, postmenopausal women enrolled in the intervention arm of a randomized controlled physical activity intervention trial. Each participant received a 16-week technology-based intervention that used the Fitbit physical activity tracker and website. The overall study goal was 150 minutes/week of moderate to vigorous intensity physical activity (MVPA) and 10,000 steps/day; however, goals were set individually for each participant and updated at Week 4 based on progress. Adherence data were collected by the Fitbit and aggregated by Fitabase. Participants also wore an ActiGraph GT3X+ accelerometer for 7 days prior to the intervention and again during Week 16.

**Results:**

The median participant logged 10 hours or more/day of Fitbit wear on 95% of the 112 intervention days, with no significant decline in wear over the study period. Participants averaged 7540 (SD 2373) steps/day and 82 minutes/week (SD 43) of accumulated “fairly active” and “very active” minutes during the intervention. At Week 4, 80% (20/25) of women chose to maintain/increase their individual MVPA goal and 72% (18/25) of participants chose to maintain/increase their step goal. Physical activity levels were relatively stable after peaking at 3 weeks, with only small declines of 8% for steps (*P*=.06) and 14% for MVPA (*P*=.05) by 16 weeks.

**Conclusions:**

These data indicate that a sophisticated, direct-to-consumer activity tracker encouraged high levels of self-monitoring that were sustained over 16 weeks. Further study is needed to determine how to motivate additional gains in physical activity and evaluate the long-term utility of the Fitbit tracker as part of a strategy for chronic disease prevention.

**Trial Registration:**

Clinicaltrials.gov NCT01837147; http://clinicaltrials.gov/ct2/show/NCT01837147 (Archived by WebCite at http://www.webcitation.org/6d0VeQpvB)

## Introduction

Physical inactivity is related to increased risk of several chronic diseases, including cardiovascular disease, stroke, Type 2 diabetes, and cancer [1-4]. Yet when assessed using objective measures, only 2-3% of middle aged and older US women are achieving physical activity levels consistent with the national recommendations [5]. Web-based technologies, including body-worn sensors and smartphone apps that utilize the phone’s onboard accelerometer, are among the most promising approaches to create scalable interventions for this serious public health problem. Analyses of theory-driven diet and physical activity interventions have shown that the component most strongly associated with successful behavior change is self-monitoring when used in combination with at least one additional self-regulatory technique (eg, goal setting, review of previously set goals, frequent behavioral feedback) [6-8]. Numerous off-the-shelf trackers are now available, many of which align well with these proven behavioral change techniques. These devices, when used within a theory-driven intervention, may therefore provide an efficient way to enable participants to improve self-regulation and adopt healthy behaviors. These trackers and apps have the added benefit of allowing investigators to obtain detailed, real-time feedback on participants’ activity level and adherence to specific physical activity goals.

Few published studies have examined the use of accelerometer-based trackers as intervention tools, particularly with regard to the aggregation and analysis of tracker data as a measure of adherence. One such device, the Fitbit, has been shown to be usable and valid for physical activity monitoring [9,10]. Published studies using the Fitbit as an intervention tool include 3 single-arm studies [11-13] and 1 randomized trial among older adults [14]. This study reports in detail the adherence of women assigned to use a clip-on Fitbit tracker as part of a low-touch physical activity intervention. Main study outcomes have been reported previously [15].

## Methods

### Participants

Participants were 25 postmenopausal, overweight or obese women assigned to a 16-week low-touch, Fitbit-based intervention as part of a randomized controlled trial. All had a body mass index (BMI) over 25.0 kg/m^2^, were regular Internet users, owned a computer or tablet with Internet access, and were able to exercise safely as determined by the Physical Activity Readiness Questionniare (PAR-Q) [16]. Procedures were approved by the University of California, San Diego (UCSD) Human Research Protections Program and written informed consent was obtained from each participant (trial registration number NCT01837147).

### Study Visits

Each participant attended 3 study visits; 2 prior to randomization and 1 at the end of the 16-week study.

### Measures

Baseline Physical Activity

The ActiGraph GT3X+, worn for 7 days prior to randomization and again at the end of the study, was used to measure baseline physical activity. This lightweight triaxial accelerometer is worn around the waist and has been validated and calibrated for use in controlled and field conditions. Standard calibration thresholds were used to aggregate data into intensity categories [17].

Baseline Demographics and Technology Use

Web-based questionnaires were used to collect demographics and technology use (items from the Pew Internet and American Life Project as well as a small number of study-specific items).

Body Mass Index

BMI (kg/m^2^) was calculated from height and weight, which were measured using standard procedures.

#### Physical Activity Adherence

The primary adherence measure during the 16-week intervention was data collected and uploaded to the Web by the Fitbit One tracker. Each participant’s Fitbit was linked to the Fitabase analytics system (Small Steps Labs, San Diego, CA, USA), which enabled the investigators to remotely monitor physical activity. Fitabase daily totals for steps and intensity-specific minutes of physical activity (PA) were downloaded at the end of the study.

### Intervention

Participants received a Fitbit One physical activity tracker, which clips to the waistband or bra or can be placed in a front pants pocket. An internal accelerometer measures motion which is then aggregated into physical activity data. Summary information (eg, steps) is available on the tracker itself and data are wirelessly uploaded to a personalized website that displays daily steps, minutes/day of activity, and a graph showing the temporal pattern of physical activity during the day and over time (eg, weeks or months).

To minimize potential barriers to navigating the technology, the project coordinator (1) set up the Fitbit account and tracker for each participant, (2) demonstrated how to download and install the Fitbit software, (3) trained the participant on the website’s self-monitoring and self-regulation features, and (4) provided the participant with an intervention handbook with study goals, information on building self-regulation skills, and detailed instructions (including screenshots) on how to install the Fitbit software. The study coordinator guided the participant through an initial goal setting process for moderate-to-vigorous intensity physical activity (MVPA) and steps and helped her develop a specific plan to achieve those goals. Study goals were 150 minute/week of MVPA and 10,000 steps/day; however, individual goals could be higher or lower. At Week 4, participants received a telephone call to evaluate progress, provide feedback, and update their personalized goals to establish targets for the remaining 12 weeks of the study. The study coordinator was logged into the participant’s Fitbit account during the call, enabling her to view an objective assessment of the participant’s progress to facilitate the goal setting process.

### Data Analysis

Analyses were completed using SAS 9.4. Baseline characteristics were compared using chi-square and *t* tests. Physical activity data (both ActiGraph and Fitbit) were adjusted for number of valid wear days and repeated measures analysis was used to assess (1) changes in physical activity and (2) revision of goals at baseline versus 4 weeks.

## Results

The intervention group consisted of 25 women (mean age 58.6 years [SD 6.5]) with a BMI of 29.2 kg/m^2^ (SD 3.8; Table 1). On the baseline ActiGraph assessment, participants were performing 24 (SD 39) minutes/week of MVPA in bouts of at least 10 minutes (the type of activity prescribed by the physical activity guidelines). None of the participants was meeting the recommended amount of 150 min/week of MVPA in bouts [18]. Eighty-four percent of participants (21/25) were daily Internet users (Table 2).

**Table 1:** Baseline characteristics of study participants (n=25).

Baseline characteristics		Mean (SD)
Age, years		58.6 (6.5)
Body mass index (kg/m^2^)		29.2 (3.8)
Non-Hispanic white, n (%)		23 (92)
College degree or higher, n (%)		14 (56)
**Moderate to vigorous physical activity** ^a^		
	Average MVPA performed in Freedson bouts (minutes/week)	24 (39)
	Total accumulated moderate to vigorous activity (minutes/week)	172 (83)
**Steps** ^a^		
	Average steps per day	5906 (1964)
	% walking ≥10,000 steps/day	4

^a^As measured by ActiGraph GT3X+ accelerometer.

### Adherence to Tracker Usage

**Figure 1:**
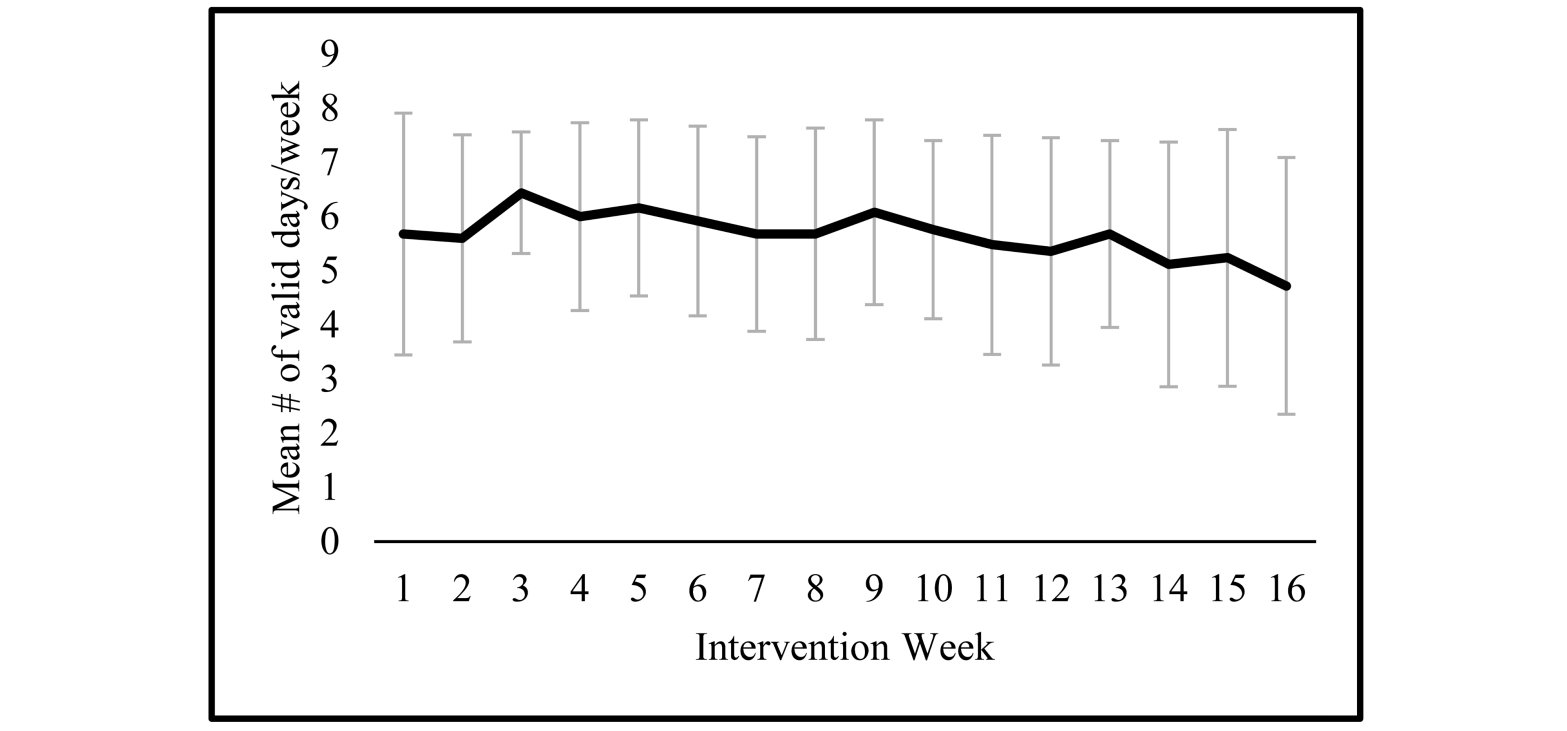
Adherence to wearing the Fitbit tracker during the 16-week intervention period among postmenopausal, overweight/obese women (N=25). Valid days are defined as those with 10 hours or more of wear time.

During the 16-week intervention, participants adhered well to wearing the tracker, with the median participant logging 10 hours/day or more of wear time on 94.6% of intervention days (106/112). Mean valid wear was 90 days (SD 22) with a range from 14 to 111 days. Adherence to tracker usage peaked at Week 3 and was maintained throughout the study period, with no significant decline in the mean number of valid wear days per week during the 16 weeks (Figure 1).

### Individualized Goal Setting

The objective feedback provided by continuous monitoring enabled the refinement of individualized goals midway through the intervention, allowing the acceleration of goals for participants who are doing well and the downward revision of goals for participants for whom the initial goal proved unrealistic. At baseline, participants set goals of 124 minute/week of MVPA (SD 34) and 8140 steps/day (SD 2224). At the 4-week goal setting call, participants slightly increased their overall MPVA goal to 143 + 70 min/week (*P*=.15) by lengthening bout duration to 32 + 13 min/bout (*P*=.01) while decreasing the bouts to 4.6 + 1.3 bouts/week (*P*=.11). Step goals marginally increased to 8660 + 2560 steps/day (*P*=.06).

**Table 2:** Technology use of study participants (n=25).

Technology use		Percentage
Daily Internet user		84
**Comfortable using computers and the Internet** ^a^		
	Neutral	4
	Somewhat or very comfortable	12
	Very comfortable	84
**Enjoys using computers and the Internet** ^b^		
	Neutral	17
	Somewhat enjoy	21
	Very much enjoy	62
**Type of primary computer**		
	Desktop	32
	Laptop	64
	Tablet	4
**Operating system of primary computer**		
	Windows	64
	Mac	36

^a^Women who responded “Very uncomfortable” or “Somewhat uncomfortable” were ineligible for this study.

^b^Women who responded “Very much do not enjoy” or “Somewhat do not enjoy” were ineligible for this study.

### Adherence to Study and Individual PA Goals

**Figure 2:**
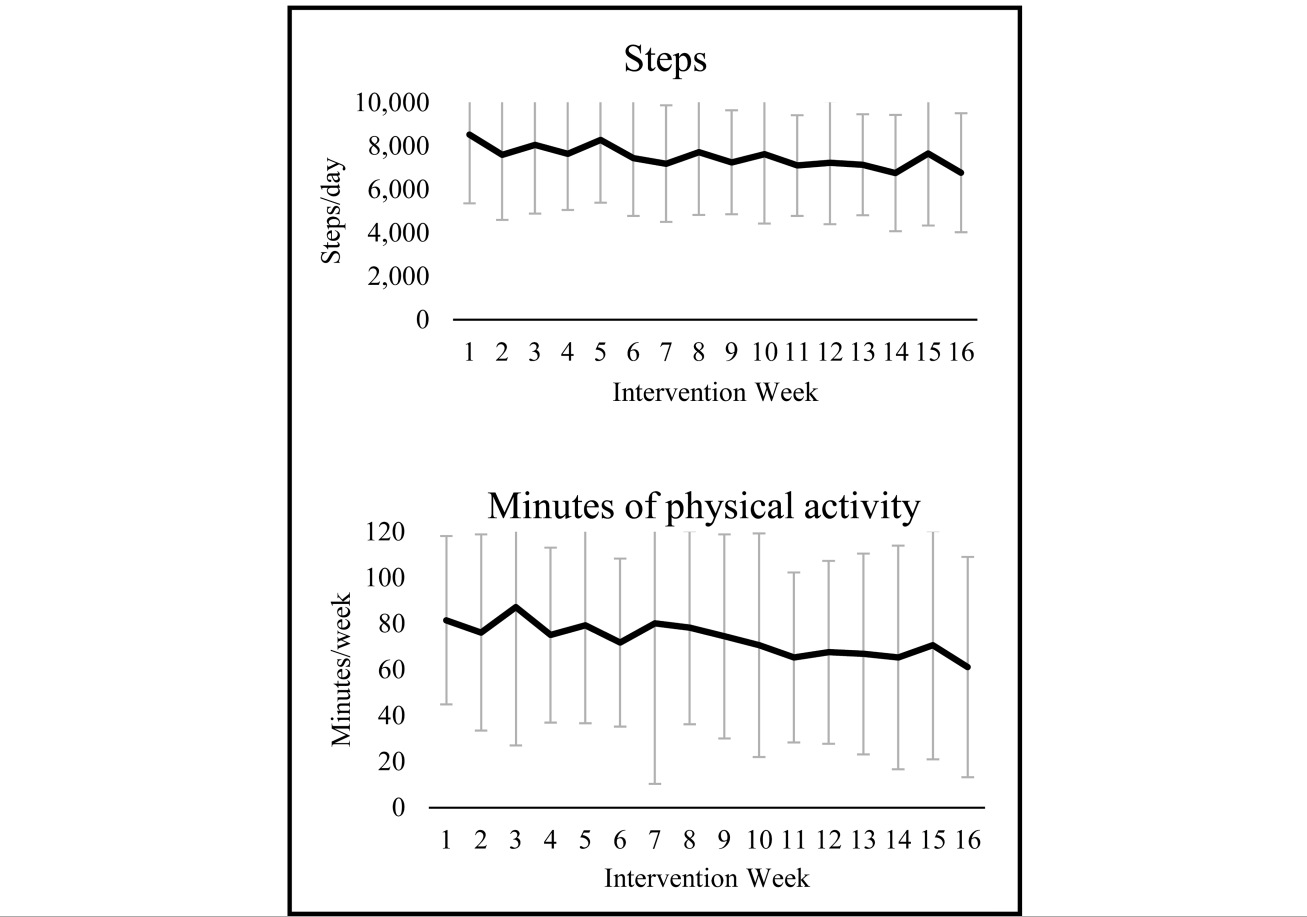
Fitbit-measured steps and minutes of “fairly or very active” physical activity during a 16-week intervention among postmenopausal, overweight/obese women (N=25).

As reported elsewhere [15], significant pre/post increases were observed on ActiGraph-measured physical activity outcomes. Physical activity adherence during the intervening 16 weeks was assessed using Fitbit data. Based on this measure, participants averaged 7540 steps/day (SD 2373) and 82 minutes/week (SD 43) of accumulated “fairly active” and “very active” minutes during the intervention period (Figure 2). Physical activity levels peaked at Week 3, but no statistically significant decline was observed through the intervention period. During Weeks 1-4 participants averaged 7922 (SD 2671) daily steps, or 98.3% of their personalized goal (mean goal: 8140 steps/day; SD 2224) and 91 minutes/week of “fairly” or “very active” minutes (SD 50), or 79.4% of their personalized goal (mean goal: 124 minutes/week; SD 34). During Weeks 5-16 participants accumulated 7395 steps/day, or 85.2% of their updated personal goal (mean goal: 8660 steps/day; SD 2561), and performed 79 minutes/week of activity (SD 45), or 59.7% of their updated goal (mean goal: 142 minutes/week; SD 70).

## Discussion

### Principal Findings

This study provides initial evidence that middle aged and older women adhere very well to wearing and using the Fitbit tracker and that it is a promising tool for continuous monitoring of physical activity adherence in this population. Unlike research accelerometers (eg, the ActiGraph), the Fitbit is suited for continuous long-term use by participants and is designed for use as a behavior change tool.

### Limitations

Limitations of this study include a specialized, nongeneralizable sample, small sample size, short study duration of 16 weeks, and inclusion of only overweight or obese individuals, who may be more likely to change in the short term. While a few studies have been published using the Fitbit as an intervention tool to provide behavioral feedback to participants [11-14], this is, to our knowledge, the first study to demonstrate the feasibility of the Fitbit for continuous monitoring of physical activity adherence by investigators.

### Future Work

More data are needed regarding the integration of consumer-based electronic sensors for physical activity promotion and monitoring within the context of behavioral medicine research. Two additional areas where research is needed are (a) studies testing the use of physical activity sensors for promotion and/or monitoring of physical activity within the health care setting and (b) additional validation studies examining the accuracy of consumer-based sensors compared with standard research measures (eg, ActiGraph) across population subgroups and for different intensities and types of physical activity. Furthermore, new models of the Fitbit and other trackers now include features such as heart rate and global positioning systems (GPS) sensors, which provide additional opportunities for assessing objective, intensity-related data on physical activity adherence. The fast pace of product development and evolution will continue to present new opportunities and challenges for behavioral mHealth research and intervention science.

## Abbreviations

BMI: body mass index
GPS: global positioning systems
MVPA: moderate-to-vigorous physical activity
PA: physical activity
UCSD: University of California, San Diego
